# A Rare Case of Severe Disseminated Pyoderma Gangrenosum of the Upper Body With Concurrent Nasopharyngeal and Inferior Orbital Wall Necrosis

**DOI:** 10.7759/cureus.98737

**Published:** 2025-12-08

**Authors:** Robert M Branstetter, Isabel E Baird, Mohammed S Rais, Danielle N Ledet, Megan N Terrebonne

**Affiliations:** 1 Medicine, Louisiana State University Health Sciences Center, New Orleans, USA; 2 Internal Medicine, Louisiana State University Health Sciences Center, Baton Rouge, USA

**Keywords:** cocaine use, levamisole-adulterated cocaine, nasal septal perforation, neutrophilic dermatosis, paraneoplastic syndrome, pathergy, polysubstance use, pyoderma gangrenosum, renal cell carcinoma

## Abstract

Pyoderma gangrenosum (PG) is a rare neutrophilic dermatosis usually linked to autoimmune or inflammatory bowel disease and typically affects the lower extremities. This case is notable for extensive upper-body, oropharyngeal, and nasal involvement in a patient without an underlying autoimmune disorder. A 56-year-old man with chronic cocaine use and stage IV renal cell carcinoma presented with rapidly progressive ulcerations of the face, neck, shoulders, and back, along with nasal septal collapse and oropharyngeal destruction. Prior biopsy demonstrated sterile neutrophilic inflammation without vasculitis, infection, or malignant infiltration, supporting PG as a diagnosis of exclusion. Imaging showed erosive sinonasal disease. He was treated with systemic and topical corticosteroids and antibiotics for concurrent methicillin-resistant *Staphylococcus aureus* (MRSA) bacteremia, as well as MRSA and *Pseudomonas aeruginosa* wound colonization. Cyclosporine was avoided because of his metastatic cancer, and both percutaneous endoscopic gastrostomy (PEG) placement and parenteral nutrition were deferred due to infection risk and concern for pathergy. With limited therapeutic options and an advanced malignancy, care transitioned to comfort measures. This case illustrates the diagnostic difficulty of PG when cocaine exposure, malignancy, and mucosal destruction coexist. The absence of vasculitides or cytopenias, combined with biopsy findings, supported PG as the unifying diagnosis. Clinicians should consider PG in atypical facial or upper-body ulcerations and recognize how comorbid conditions may restrict standard treatment.

## Introduction

Pyoderma gangrenosum (PG) is a rare, inflammatory skin disorder characterized by neutrophilic dermatosis and tender, ulcerated cutaneous lesions, most commonly found on the lower extremities [1]. It is often associated with systemic conditions, such as rheumatoid arthritis, ulcerative colitis, and Crohn’s disease [2]. Although the pathogenesis remains incompletely understood, immune dysregulation and aberrant neutrophil activity are believed to play key roles [3]. Classic PG usually presents as a painful ulcer on the legs, whereas disseminated, upper-body, mucosal, or head and neck involvement is uncommon and typically reflects atypical or severe variants [4].

PG remains a diagnosis of exclusion, and distinguishing it from infection, vasculitis, malignancy-related ulceration, or drug-induced vasculopathy can be challenging, especially when multiple risk factors coexist [1]. Cocaine and levamisole exposure have increasingly been recognized to cause PG-like ulcerations, including cribriform lesions and destructive midline changes, adding further diagnostic complexity in affected patients [4]. Atypical PG, particularly when associated with malignancy or cocaine use, may also respond poorly to standard immunosuppressive therapy, which can complicate management [4].

This case illustrates such a diagnostic challenge, as the patient had metastatic cancer, chronic cocaine use, extensive ulceration, and progressive facial tissue loss. Here, we describe a unique case of biopsy-confirmed PG in a patient with no underlying autoimmune or inflammatory bowel disease, presenting with extensive full-thickness ulcerations involving the upper body and face.

## Case presentation

We present a case of a 56-year-old male with a history of polysubstance use (alcohol and cocaine), hypertension, an unclear history of seizures, and stage IV clear cell renal carcinoma with pulmonary metastases, who was transferred to our Emergency Department from an outside hospital.

The patient’s oncologic and dermatologic course is summarized chronologically as follows. A renal mass concerning for clear cell renal carcinoma was first identified in March 2023 and confirmed by core needle biopsy in October 2023, at which time imaging demonstrated multiple pulmonary nodules consistent with metastatic disease. Thibodaux Oncology recommended systemic therapy with pembrolizumab and axitinib, which he subsequently initiated. He completed nine cycles before developing ulcerated paraneoplastic-appearing skin lesions in June 2024.

Dermatology obtained biopsies of these lesions in July 2024. Histopathology demonstrated acute inflammation with abscess formation and no evidence of malignancy. Immunostains, including AE1/AE3, CD10, and RCC markers, were negative, excluding metastatic renal cell carcinoma. Special stains (Gomori methenamine silver (GMS) and periodic acid-Schiff (PAS)) were negative for fungal organisms, and the acid-fast bacillus (AFB) stain showed only nonspecific background staining. No organisms were identified, and there was no vasculitis. Although the findings were nonspecific, the biopsy demonstrated sterile neutrophilic inflammation and excluded infection and malignancy. When combined with the rapidly progressive ulcers with violaceous, undermined borders, these findings supported a diagnosis of PG as a diagnosis of exclusion.

Systemic therapy was held for approximately 3.5-4 months without improvement in his skin lesions, and dermatology ultimately felt the lesions were unlikely to be treatment-related. He was lost to follow-up between June and November 2024. Upon re-evaluation in November 2024, he had persistent ulcerations, although oncologic imaging showed improvement in his renal and pulmonary disease, so pembrolizumab and axitinib were restarted.

Progressive facial swelling was first documented in March 2025, several months after systemic therapy had been resumed. He was admitted to an outside hospital at that time for a tension pneumothorax, which was attributed to either a bronchopleural fistula related to his metastatic disease or possible pulmonary involvement of PG. He was discharged on a prednisone taper, and records indicate he remained on chronic prednisone 10 mg daily thereafter. The exact onset and tempo of nasal and oropharyngeal tissue loss are not clearly recorded, but CT imaging in April 2025 demonstrated complete opacification of the bilateral mastoid air cells without evidence of recurrent malignancy. By May 5, 2025, he had completed 16 cycles of pembrolizumab.

**Figure 1:**
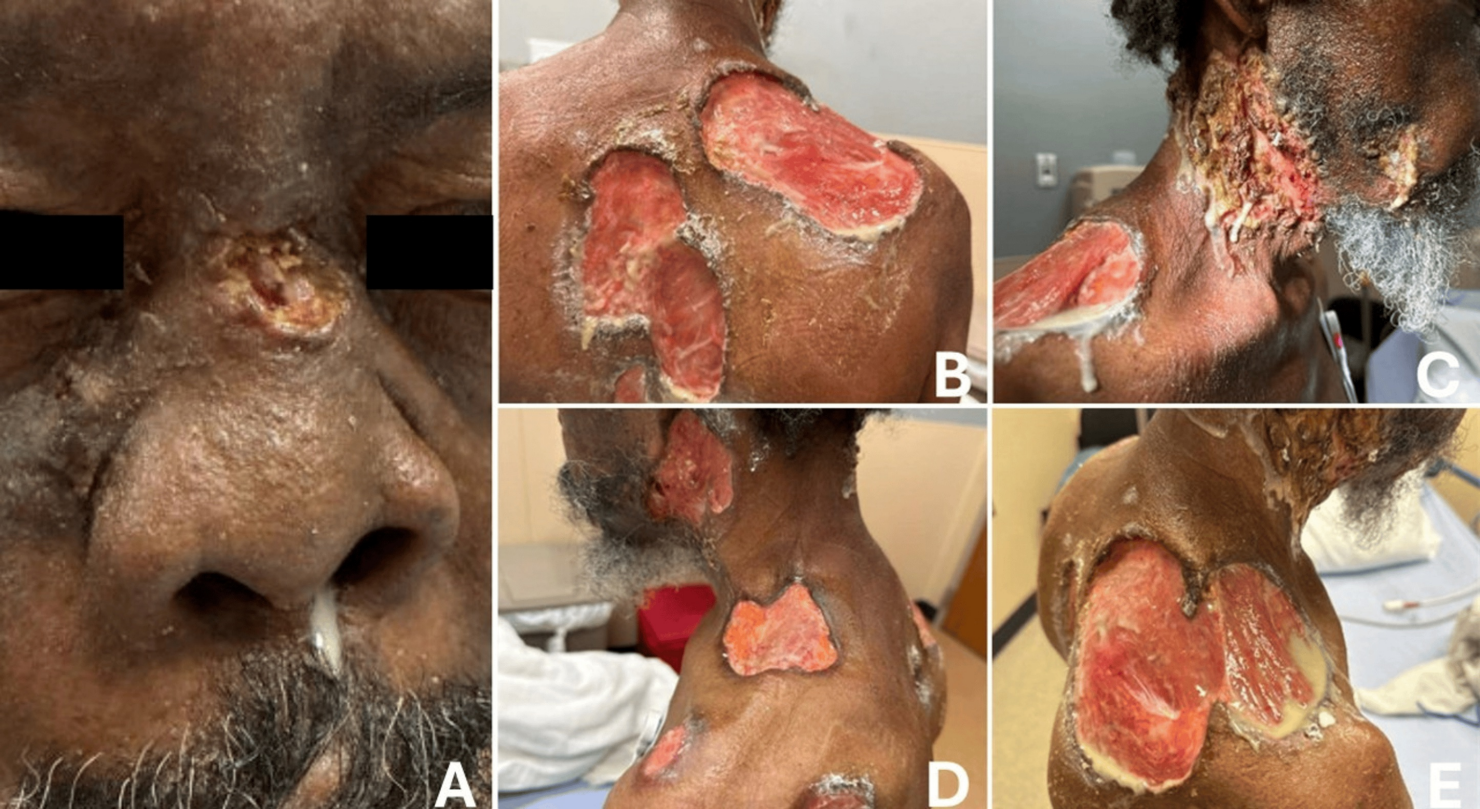
Clinical presentation of pyoderma gangrenosum (PG). (A) Ulceration with collapse of the nasal bridge and crusting of the nasal cavity, demonstrating severe mucocutaneous involvement that may overlap with cocaine-associated midline destruction. (B) Full-thickness ulcerations of the right posterior shoulder and back with undermined borders characteristic of pyoderma gangrenosum. (C) Necrotic ulcerations involving the right lateral neck and shoulder with purulent drainage, reflecting secondary infection superimposed on PG lesions. (D) Ulcerations over the left lateral neck and shoulder showing violaceous, undermined borders consistent with PG. (E) Extensive ulcerative lesions with exposed underlying tissue on the right posterior shoulder, highlighting the depth and severity typical of disseminated PG.

The patient presented to our hospital on May 11, 2025, with decreased oral intake due to facial wounds, nasal bridge collapse, and purulent nasal drainage. He reported that liquids taken by mouth drained through his nose, leading to severe weight loss and malnutrition. Primary examination revealed multiple purulent, ulcerating, full-thickness wounds of the neck, shoulders, and back, characterized by red inflammatory plaques with violaceous, undermined borders and irregular serpiginous edges, several exposing underlying muscle (Figure 1). He also had extensive destruction of the nasal bridge, midface, and bilateral lateral neck with purulent drainage and visible underlying soft tissue (Figure 1). Based on available records, the exact rate of progression is uncertain, though the lesions evolved between June 2024 and this hospitalization. He appeared cachectic with a BMI of 17 kg/m². His speech was garbled due to oropharyngeal destruction. He was afebrile and hemodynamically stable on admission.

Laboratory studies showed leukocytosis to 18.3 × 10⁹/L, elevated absolute neutrophils (11.7 × 10³/µL), erythrocyte sedimentation rate (ESR) of 211.3 mg/L, and an IntelliSep Band 2, IntelliSep Index (ISI) of 5.7. Anti-neutrophil cytoplasmic antibody (ANCA) status was obtained during this admission. Blood cultures grew methicillin-resistant *Staphylococcus aureus* (MRSA), likely secondary to his multiple draining wounds. Wound cultures (May 12, 2025) were positive for MRSA and *Pseudomonas aeruginosa*. Computed tomography (CT) of the sinuses, neck, and face (May 11, 2025) showed osseous erosion of the palate, absence of the nasal septum, and diffuse right-sided soft-tissue defects involving the superior neck, right cheek, and nasopharynx.

Otolaryngology performed bedside nasal endoscopy with debridement and saline irrigation on May 12, which improved his facial swelling and nasal airway patency. Initial endoscopy showed the nasal cavity completely obstructed by crusting; however, after debridement over the next two days, the underlying mucosa appeared healthy. He was placed on hourly nasal saline irrigations to prevent re-crusting. The sinonasal findings were suspected to represent a process distinct from his cutaneous lesions and were believed primarily related to chronic intranasal cocaine use.

Oral solumedrol and topical corticosteroids were started on the day of admission despite concurrent bacteremia, and intravenous ciprofloxacin and linezolid were initiated for MRSA bacteremia and wound infection. Follow-up blood cultures obtained two days later showed no growth. Cyclosporine was deferred due to its potential to accelerate the progression of clear cell renal carcinoma. His skin lesions stabilized without further progression during hospitalization.

His severe facial swelling and oropharyngeal deformity prevented placement of a feeding tube. A PEG tube was not attempted due to the risk of pathergy. Total parenteral nutrition was considered but ultimately deferred due to the risk of worsening bacteremia and the patient’s care goals. Given his wishes, progressive decline, nutritional intolerance, and terminal metastatic cancer, care was transitioned to comfort-focused management. He was discharged to hospice on hospital day 12 with a planned three-week steroid taper and instructions to complete the remaining antibiotic course.

## Discussion

We report this patient because his severe presentation of PG is not well-described in existing literature. Although up to 50% of patients with PG have an associated systemic inflammatory disease (e.g., inflammatory bowel disease, rheumatoid arthritis, hematologic malignancy) [5], our patient had no such diagnoses. Instead, he had other comorbidities, including polysubstance use and solid-organ malignancy, which may have served as provocative factors for his disease.

Certain drugs are a well-known cause of PG. Of the drugs that precipitate drug-induced PG, cocaine is the most common. This phenomenon is thought to be due to levamisole, a veterinary anthelmintic frequently used as a cocaine adulterant to increase product bulk, which has been described to be present in up to 80% of cocaine samples [4,6]. Cocaine-induced PG (CIPG) has a wide array of presentations and may be difficult to diagnose serologically. One of the most consistent positive serologic studies seen in cocaine-induced PG is the presence of p-ANCA antibodies [4].

Unfortunately, the ANCA status of this patient during his hospital admission and throughout previous medical workup is unavailable. Although ANCA serology testing can help characterize cocaine- or levamisole-associated autoantibody patterns, it is not required for the diagnosis of PG and is most useful for excluding vasculitic mimics [4,7]. Although ANCA positivity is frequently reported in cocaine-associated PG, it is neither sensitive nor specific enough to reliably distinguish CIPG from classic PG, and its primary utility remains in excluding ANCA-associated vasculitides. [4]. In addition, the patient's previous biopsy did not show vasculitis or thrombotic changes, making ANCA supportive but not essential for ruling out vasculitides in this case.

Our patient's previous histopathology from July 2024 provided important support for the diagnosis of PG. The biopsy demonstrated acute inflammation with abscess formation and a dense neutrophilic infiltrate, without evidence of leukocytoclastic vasculitis, thrombotic vasculopathy, fungal or mycobacterial organisms, or malignancy. Immunostains, including AE1/AE3, CD10, and RCC markers, were negative, effectively excluding metastatic renal cell carcinoma as a cause of the ulcerations. Special stains (PAS and GMS) were negative for fungal organisms and mycobacteria. The absence of vasculitis is particularly notable, as it argues against levamisole-associated vasculopathy, which is a key mimic of PG in cocaine users.

Additionally, levamisole-associated vasculopathy frequently presents with leukopenia or neutropenia, which is considered one of its hallmark systemic findings [4]. In contrast, our patient’s complete blood count on admission showed no leukopenia or neutropenia; instead, he demonstrated mild leukocytosis with elevated absolute neutrophils (11.7 × 10³/µL), consistent with an inflammatory response rather than levamisole toxicity. The absence of cytopenias therefore argues against levamisole-associated vasculitis while remaining consistent with cocaine-induced PG. Wound cultures on admission grew MRSA and *P. aeruginosa*, which likely represent secondary colonization or superinfection rather than a primary infectious etiology. Secondary bacterial growth is common in PG due to the presence of open ulcerations, and the clinical findings in this case were not consistent with necrotizing infection. Infection must be excluded as it can resemble PG, and because initiating immunosuppression in an untreated infection may lead to further progression.

When compared to the current literature, our patient has unique clinical features of his PG presentation that may correlate with cocaine-induced PG. In two cases presented by Lemieux et al., both patients had an extensive history of cocaine misuse and presented with multiple diffuse ulcerative lesions. In both cases, the wounds were largely treatment resistant and tended to resolve with cessation of cocaine use, indicating a strong temporal association between cocaine use and PG [4]. While the temporal association between his cocaine use and PG severity is unclear, his PG also failed to improve despite chronic prednisone use after his March 2025 hospitalization. However, his regimen consisted of 10 mg daily, which is substantially below the therapeutic dosing typically required for active PG, so the incomplete response may reflect subtherapeutic treatment rather than a cocaine-related effect.

Additionally, head and neck involvement in PG occurs in 5% of all cases, but has been reported in up to 75% of cocaine-induced PG [4]. The lesions in our case were primarily located on the upper half of the patient’s body, including his face, large sections of his back, and shoulders. This presentation is atypical, as classic PG usually presents on the lower extremities [8]. Furthermore, nasal PG can involve the nasal septum and result in saddle nose deformity, as seen in our patient [9]. A similar case reported by Sehgal et al. describes a patient with cocaine use disorder that manifested as cutaneous and nasal mucosa PG, as well as a perforated septum and pneumonitis [10]. Our patient shared similarities with their patient’s reported lesions, as they were also located in the nose and on the upper back. The similarities between these previously reported cases and our patient suggest that cocaine-induced pyoderma gangrenosum is a plausible etiology.

However, interpretation of the patient’s nasopharyngeal destruction presents an additional diagnostic challenge. Chronic intranasal cocaine use is a well-recognized cause of midline destructive lesions due to ischemia and mucosal necrosis, often leading to septal perforation and collapse of the nasal bridge [11]. PG, although far less commonly involving the nasal mucosa, has also been reported to cause septal erosion and nasal deformity in atypical cases [9]. In our patient, the severity of nasopharyngeal tissue loss, combined with his long-standing cocaine use and the presence of biopsy-proven neutrophilic dermatosis elsewhere, suggests that his nasopharyngeal destruction is most likely multifactorial. Both cocaine-related ischemic injury and mucosal PG may have contributed to the extent of tissue loss, but the relative contribution of each cannot be definitively determined.

Another etiology of PG described in the current literature is PG occurring as a paraneoplastic process associated with malignancy. PG has been reported as a paraneoplastic syndrome in both hematologic and solid-organ cancers [12]. Our patient’s stage IV clear cell renal carcinoma with pulmonary metastases raises this possibility. In a retrospective review of five patients with PG and solid-organ malignancy, PG and tumor onset occurred within three months of each other, and recurrent PG often paralleled tumor recurrence [13]. Additionally, a paraneoplastic PG could explain the patient’s treatment resistance. In a case reported by Regnier-Rosencher et al., a male patient presented with treatment-refractory PG of the lower extremity and was later diagnosed with renal cell carcinoma, for which he underwent partial nephrectomy. After the nephrectomy, the patient’s PG resolved with corticosteroid creams [14]. Another case report by Cosgarea et al. depicts the successful treatment of PG in a patient with renal cell carcinoma with the use of ustekinumab after radical nephrectomy was performed [15]. These cases highlight the potential interplay of PG and malignancy, as well as the importance of malignancy screening in patients with refractory PG. Given that our patient developed PG shortly after initiating cancer therapy, this temporal relationship may suggest a paraneoplastic association. However, a definitive correlation is difficult to establish, as the patient was intermittently lost to follow-up throughout his oncologic course, and the status of his lesions was unknown during this time. Furthermore, given our patient's diagnosis of stage IV clear cell renal carcinoma with pulmonary metastases, surgical nephrectomy was not deemed a viable treatment option.

In summary, the integration of this patient’s clinical presentation, histopathology, culture results, hematologic profile, oncologic history, and substance use strongly supports a diagnosis of PG rather than vasculitis, necrotizing infection, or malignancy-related ulceration. Both cocaine exposure and underlying renal cell carcinoma may have contributed to this patient’s disease, as each has been reported in the literature as a trigger of atypical or treatment-refractory PG. Based on the available clinical, histopathologic, and laboratory data, we cannot determine whether his PG is predominantly cocaine-associated or paraneoplastic, and it is possible that both mechanisms played a role. However, the absence of vasculitis on biopsy, the lack of cytopenias, and the neutrophilic dermatosis support PG as the unifying diagnosis rather than primary levamisole-associated vasculitis, necrotizing infection, or direct malignant infiltration. Overall, his presentation most likely reflects severe PG arising in a multifactorial context, highlighting the diagnostic complexity of PG in patients with concurrent malignancy and substance use.

## Conclusions

This case highlights the significant diagnostic and therapeutic challenges posed by severe PG in a patient with multiple overlapping risk factors and competing clinical explanations for his findings. The combination of profound upper-body ulcerations, extensive nasopharyngeal destruction, chronic cocaine use, and metastatic renal cell carcinoma created a clinical picture in which infection, vasculitis, malignancy, drug-induced injury, and PG all remained plausible at presentation. In this context, the biopsy demonstrating sterile neutrophilic inflammation without vasculitis or malignant infiltration, the absence of cytopenias, and the exclusion of fungal, mycobacterial, and bacterial primary infection were critical for arriving at PG as the unifying diagnosis. The severity of his nasopharyngeal involvement illustrates the overlap between mucosal PG and cocaine-associated midline destructive lesions and highlights the diagnostic ambiguity that arises when ischemic cocaine-related necrosis and neutrophilic mucosal disease coexist.

Management was equally complex. His refractory ulcerations, severe malnutrition, bacteremia, and ongoing need to avoid pathergy significantly limited therapeutic options. Cyclosporine, a standard PG therapy, was avoided due to concern for accelerating the progression of his metastatic renal cell carcinoma. Percutaneous endoscopic gastrostomy (PEG) tube placement, typically used for nutrition in patients with severe facial injury, was contraindicated due to the risk of PG pathergy, while total parenteral nutrition carried unacceptable infectious risk in the setting of MRSA bacteremia. Although corticosteroids stabilized his cutaneous disease, his worsening nutritional intolerance and underlying terminal malignancy precluded further escalation of immunosuppressive therapy. This case illustrates how PG can become exceptionally difficult to diagnose and manage when occurring alongside cocaine use, malignancy, infection, and severe catabolic state. It emphasizes the need for a thorough exclusion of mimicking diseases, careful integration of histopathology and clinical course, and individualized treatment planning that accounts for oncologic status, infection risk, nutritional compromise, and the potential for pathergy. Although the relative contributions of cocaine exposure and metastatic renal cell carcinoma cannot be fully delineated, both mechanisms are well-described triggers of atypical PG and likely contributed to the severity and refractory nature of the disease in this patient. Ultimately, this patient’s presentation reinforces that PG in medically complex individuals is often multifactorial and may require both diagnostic humility and therapeutic restraint.
